# Identification of New FG-Repeat Nucleoporins with Amyloid Properties

**DOI:** 10.3390/ijms24108571

**Published:** 2023-05-10

**Authors:** Lavrentii G. Danilov, Xenia V. Sukhanova, Tatiana M. Rogoza, Ekaterina Y. Antonova, Nina P. Trubitsina, Galina A. Zhouravleva, Stanislav A. Bondarev

**Affiliations:** 1 Department of Genetics and Biotechnology, St. Petersburg State University, 199034 St. Petersburg, Russia; 2 St. Petersburg Branch, Vavilov Institute of General Genetics, Russian Academy of Sciences, 194064 St. Petersburg, Russia; 3 Laboratory of Amyloid Biology, St. Petersburg State University, 199034 St. Petersburg, Russia

**Keywords:** nucleoporins, amyloids, ArchCandy, C-DAG, evolution, FG-repeats

## Abstract

Amyloids are fibrillar protein aggregates with a cross-β structure. More than two hundred different proteins with amyloid or amyloid-like properties are already known. Functional amyloids with conservative amyloidogenic regions were found in different organisms. Protein aggregation appears to be beneficial for the organism in these cases. Therefore, this property might be conservative for orthologous proteins. The amyloid aggregates of the CPEB protein were suggested to play an important role in the long-term memory formation in *Aplysia californica*, *Drosophila melanogaster*, and *Mus musculus*. Moreover, the FXR1 protein demonstrates amyloid properties among the Vertebrates. A few nucleoporins (e.g., yeast Nup49, Nup100, Nup116, and human Nup153 and Nup58), are supposed or proved to form amyloid fibrils. In this study, we performed wide-scale bioinformatic analysis of nucleoporins with FG-repeats (phenylalanine–glycine repeats). We demonstrated that most of the barrier nucleoporins possess potential amyloidogenic properties. Furthermore, the aggregation-prone properties of several Nsp1 and Nup100 orthologs in bacteria and yeast cells were analyzed. Only two new nucleoporins, *Drosophila melanogaster* Nup98 and *Schizosaccharomyces pombe* Nup98, aggregated in different experiments. At the same time, *Taeniopygia guttata* Nup58 only formed amyloids in bacterial cells. These results rather contradict the hypothesis about the functional aggregation of nucleoporins.

## 1. Introduction

Amyloids are fibrillar protein aggregates with cross-β structure (for review, see [1,2,3,4,5,6]). Amyloids were discovered as protein deposits associated with different diseases. Nowadays, more than 50 different proteins and peptides are known to form amyloid and amyloid-like aggregates in the case of several human diseases. Prominent among them are Aβ (Alzheimer’s disease), huntingtin (Huntington’s disease), α-synuclein (Parkinson’s diseases), and amylin (type 2 diabetes) ([1,3,4,5]). The functional role of several amyloids was demonstrated. The Het-s amyloid aggregates are known to play a key role in the heterokaryon incompatibility reaction in *Podospora anserina* ([7]). Aggregation of RIPK1 and RIPK3 is an essential step for intracellular signal transduction ([8]). A number of proteins that compose or are linked with the cell wall form were supposed to be amyloids, among them are Bgl2, Gas1, Toh1, and Ygp1 from *Saccharomyces cerevisiae*; chaplins from *Streptomyces coelicolor*; hydrophobins from different species; and structural components of bacterial biofilms (for review see [4,5]). FXR1 amyloid aggregates were found in the brains of rats [9] and several other vertebrates [10] and were proposed to play a role in RNA protection [9]. The discovery of thioflavin S and Congo Red-positive structures in oocytes of *Gallus gallus* and eggshells of *Drosophila melanogaster* allowed authors to suppose the existence of functional, however, still unknown, amyloids in female reproductive cells [11].

The nuclear pore complex (NPC) is a macromolecular assembly composed of different nucleoporins (Nups). These proteins can be divided into three groups: membrane, scaffold, and barrier nucleoporins [12]. Barrier Nups are presented in the inner ring of the NPC and fill the central channel by flexible regions containing phenylalanine–glycine repeats (FG-repeats) [13,14]. Some of these proteins can form amyloid or amyloid-like aggregates. One of the yeast Nups, Nsp1, forms stable hydrogels, and many other yeast Nups aggregate in vivo [15,16,17,18,19]. It was proposed that amyloid aggregates play an important role in the formation of hydrogel in the nuclear pore. This gel performs a barrier function and controls the transport through the pore [18]. Recent studies support an opposite point of view that a selective barrier in NPC is maintained by liquid-like phase separation of FG-repeat regions of Nups [20,21]. Thus, the biological role of Nups amyloid aggregation may be controversial.

## 2. Results

### 2.1. Several Nups with FG-Repeats Contained Fragments with Conservative Amyloidogenic Properties

**Figure 1:**
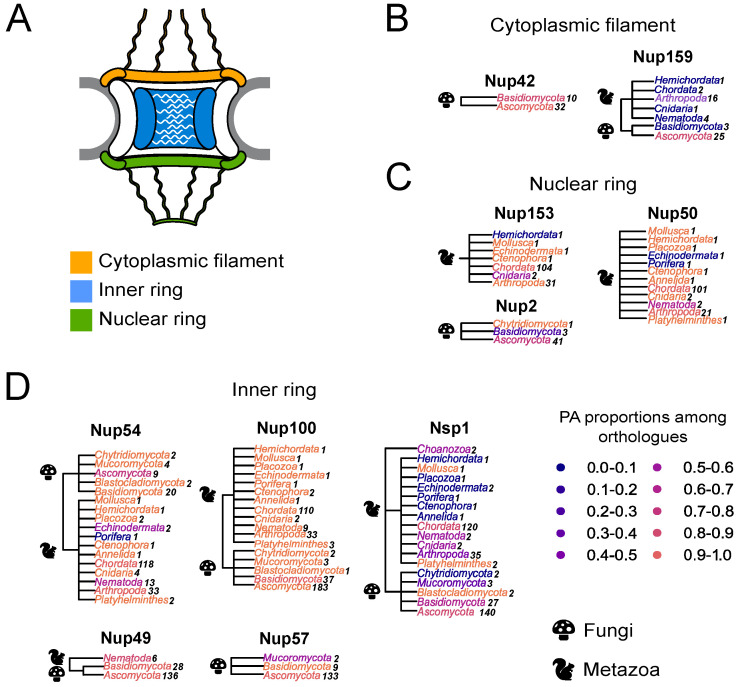
Many Nups are potential amyloids: (**A**) Schematic representation of the NPC. (**B**–**D**) The distribution of potential amyloids among FG-Nups. The NCBI taxonomy is used to represent trees. The proportion of potential amyloids is shown by the color gradient, where the maximum corresponds to orange and the minimum to dark blue. Each phylum is colored according to the proportion of amyloidogenic proteins in it. Kingdoms are mentioned with icons. A number of analyzed sequences is presented next to the phylum. Trees are grouped according to Nups’ localization in the nuclear pore complex: (**B**)—Nups of the cytoplasmic ring; (**C**)—Nups of the inner ring; (**D**)—Nups of the nuclear ring.

Different Nups with FG-repeats were suggested to form amyloid aggregates [15,16,17,18,19]. Based on these assumptions, we decided to perform a large-scale bioinformatic screening of amyloid proteins among other Nups with FG-repeats. Different programs have been developed to predict the ability of proteins to form amyloid structure. Among them, ArchCandy was shown to be one of the most accurate tools [22,23,24]. The sequences of ortholog proteins for known FG Nups were taken from the EggNOG database (accession date: 28 November 2020) and analyzed with ArchCandy. The protein was considered as amyloidogenic if it contained at least one β-arch in its unstructured part. The rates of potential amyloids were calculated for ortholog groups from different phyla (Figure 1). The results demonstrate that most of the analyzed proteins are amyloidogenic.

**Figure 2:**
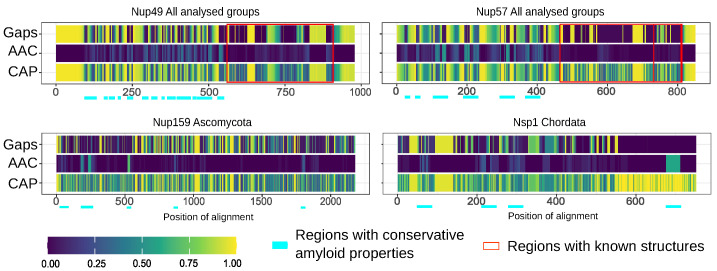
Several nucleoporins possess regions with conservative amyloidogenic properties: Gaps—frequency of gaps in the position of alignment. AAC (Amino Acids Conservatism)—rate of most frequent amino acids. CAP (Conservatism of Amyloidogenic Properties)—frequency of cases when corresponding position is located inside amyloidogenic region. Regions with known structures are highlighted by red frames. Light cyan corresponds to potential conservative amyloidogenic regions.

**Figure A1:**
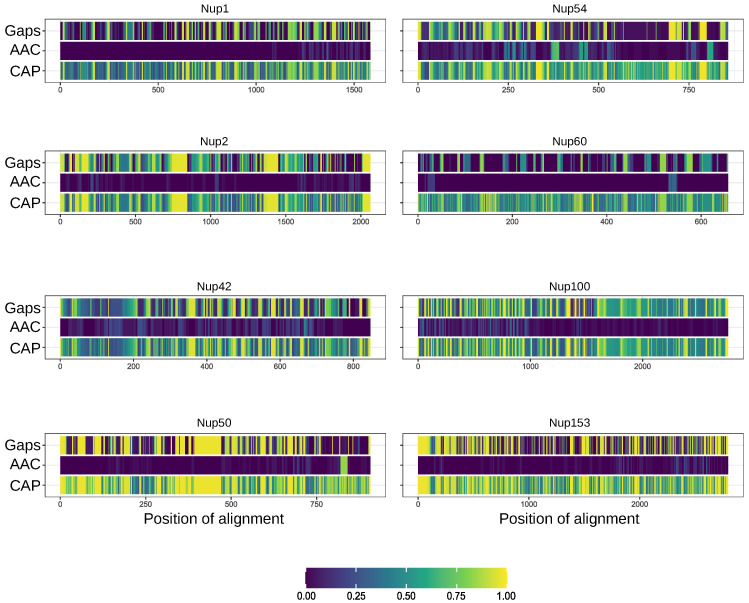
Nucleoporins lack conservative regions with amyloidogenic features. Gaps—frequency of gaps in the position of alignment. AAC (Amino Acids Conservatism)—rate of most frequent amino acids. CAP (Conservatism of Amyloidogenic Properties)—frequency of cases when corresponding position is located inside amyloidogenic region.

Furthermore, we compared the localization of predicted amyloidogenic regions in different proteins. We performed the random sampling for overrepresented sequences to avoid the bias linked to the different numbers of proteins in distinct taxonomic groups followed by the multiple sequence alignment of orthologs. Then, the information about amyloidogenic regions within corresponding proteins was superimposed on the alignment. Finally, for each alignment position, we calculated the (i) frequency of gaps, (ii) rate of most frequent amino acids (Amino Acids Conservatism or AAC in Figure 2), and (iii) frequency of cases when the corresponding position is located inside the amyloidogenic region (Conservatism of Amyloidogenic Properties or CAP in Figure 2) (see the Materials and Methods Section for details). The analysis revealed several regions with conservative amyloidogenic properties (high CAP and low rate of gaps) in Nup49 and Nup57 orthologs. For Nup159 and Nsp1 orthologs, analogous regions were found only for Ascomycota and Chordata (Figure 1 and Figure 2 and Appendix A Figure A1). In several cases, the conservation of amyloidogenic properties in these regions is even higher than the similarity of protein sequences. Noteworthily, all of these proteins are located within the nuclear pore channel and contain FG-repeats.

### 2.2. A Few Orthologs of Yeast Nsp1, Nup100, and Nup145 Proteins Can Form Amyloids in the C-DAG System

Several studies show that yeast Nsp1 and Nup100 are potential amyloids [15,16,17]. Both proteins aggregate in yeast cells [15,17]. Nsp1 forms a hydrogel with interchain β-sheets in vitro [25,26] and aggregates stained with ThT [15]. Fibrillar aggregates of Nup100 were stained with ThT in vitro [17]. Based on these data, we decided to check the ability of their orthologs to form amyloid aggregates. We created a collection of pDONR221 plasmids bearing fragment coding amyloidogenic regions of corresponding proteins (Table 1).

**Figure 3:**
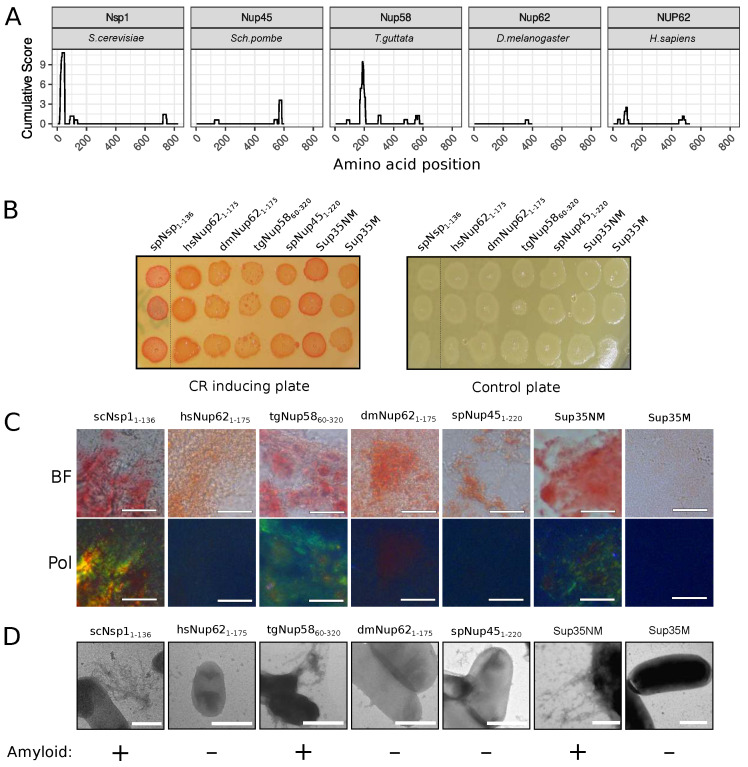
The Nup58 protein from *T. guttata* possess amyloid properties: (**A**) Amyloidogenic regions in nucleoporins. The ArchCandy Cumulative Score presented in plots reflects the ability of the protein to form β-arches. (**B**) Colonies’ color of bacteria, overproducing the amyloidogenic regions of nucleoporins, on the medium containing Congo Red (CR-inducing plate). The control plate, containing only antibiotics, was used to check the amount of plated cells. The construction names are listed in Table 1. (**C**). Microphotographs of bacterial cells from CR-inducing plate in transmitted (BF) and polarized light (Pol). The scale bar equals 20 μm. (**D**) TEM microphotographs of the cells from plates were presented on panel (**B**). Plasmids coding Sup35NM and Sup35M were used as positive and negative controls in C-DAG experiments, respectively. The scale bar equals 1 μm. The conclusion about amyloid properties of a protein based on different experiments is presented below panel (**D**).

**Figure 4:**
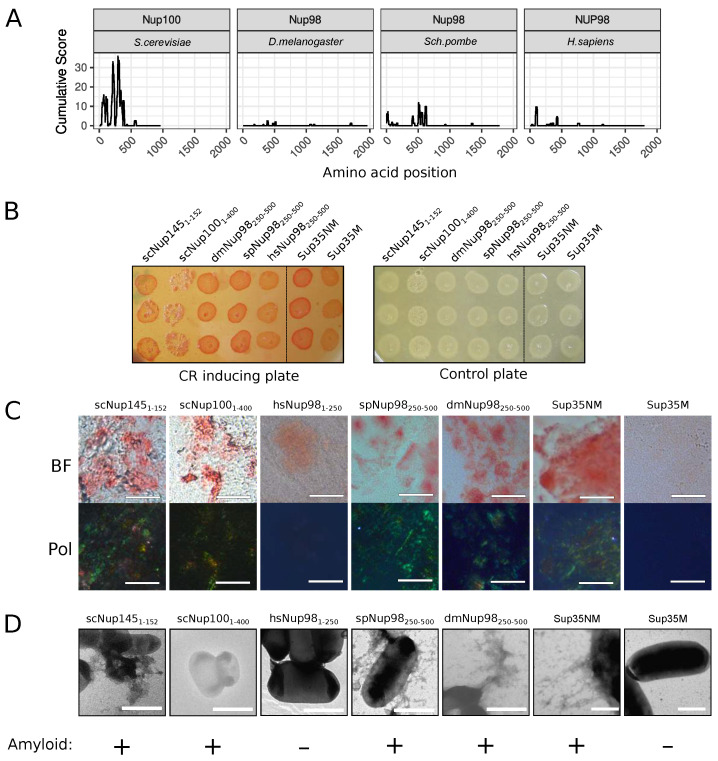
Several Nup100 and Nup145 orthologs from different organisms demonstrate amyloidogenic properties in the C-DAG system: (**A**) Amyloidogenic regions in nucleoporins. The ArchCandy Cumulative Score presented on plots reflects the ability of the protein to form β-arches. (**B**) Colonies’ color of bacteria, overproducing the amyloidogenic regions of nucleoporins, on the medium containing Congo Red (CR inducing plate). The control plate, containing only antibiotics, was used to check the amount of plated cells. The construction names are listed in Table 1. (**C**) Microphotographs of bacterial cells from the CR-inducing plate in transmitted (BF) and polarized light (Pol). The scale bar equals 20 μm. (**D**). TEM microphotographs of the cells from plates were presented on panel (**B**). The same controls as in Figure 3 (Sup35NM and Sup35M) were presented. The scale bar equals 1 μm. The conclusion about amyloid properties of a protein based on different experiments is presented below panel (**D**).

The C-DAG (curli-dependent amyloid generator) system was previously developed as an approach for testing amyloid properties of proteins. The formation of amyloid aggregates is detected by (i) the red color of cells grown on the media with Congo Red dye, (ii) their apple-green birefringence in cross-polarized light, and (iii) the appearance of fibrillar aggregates on the cell surface. The amyloidogenic (NM) and nonamyloidogenic (M) regions of Sup35 were used as positive and negative controls, respectively [27]. Potential amyloidogenic regions of tested proteins are shown in Figure 3A. The overproduction of the Nsp1 amyloidogenic region led to the red colony color of bacteria on the CR-inducing plate (Figure 3B). These cells demonstrated apple-green birefringence in polarized light (Figure 3C) and contained protein fibrils on their surface (Figure 3D). This result supported the data that the Nsp1 can form amyloid aggregates [15] and demonstrated the accuracy of the C-DAG system. Among other analyzed proteins, only Nup58 of *T. guttata* (tgNup58_60–320_) demonstrated the same properties as Nsp1 and Sup35NM (Figure 3). Thus, we concluded that only Nsp1 of *S. cerevisiae* and Nup58 of *T. guttata* are amyloids among the analyzed Nsp1 orthologs. We conducted analogous experiments for several homologs of yeast Nup100 protein (Figure 4). Our results provide additional evidence that yeast Nup100 and Nup145 are amyloids [15,17]. Moreover, we found that Nup98 proteins from *D. melanogaster* and *S. pombe* (spNup98_250–500_ and dmNup98_250–500_) demonstrated amyloid properties in the C-DAG system (Figure 4). Our results also confirm that overproduction of large proteins in this system may affect cell viability. The Nup100_1–400_ fragment was the largest one in our analysis and only its overproduction led to a decrease in cell growth (Figure 4B).

### 2.3. The [PIN^+^] Factor Does Not Affect the Aggregation of Nucleoporins of Different Species in the Yeast S. cerevisiae

In order to verify that the C-DAG negative results are nonspecific for the model system, we analyzed the ability of the same protein fragments to aggregate in yeast. Different factors may affect protein aggregation in yeasts; the [*PIN^+^*] prion is one of them [28,29]. The Rnq1 protein forms amyloid aggregates in cells with this prion [28,29,30]. The remarkable feature of these aggregates is their ability to promote or affect the aggregation of other amyloid-prone proteins. For example, the [*PIN^+^*] factor is required for the appearance of Sup35 and Nup100 aggregates [17,29,31] and also modulates the toxicity of heterologous huntingtin protein in yeasts [32].

**Figure 5:**
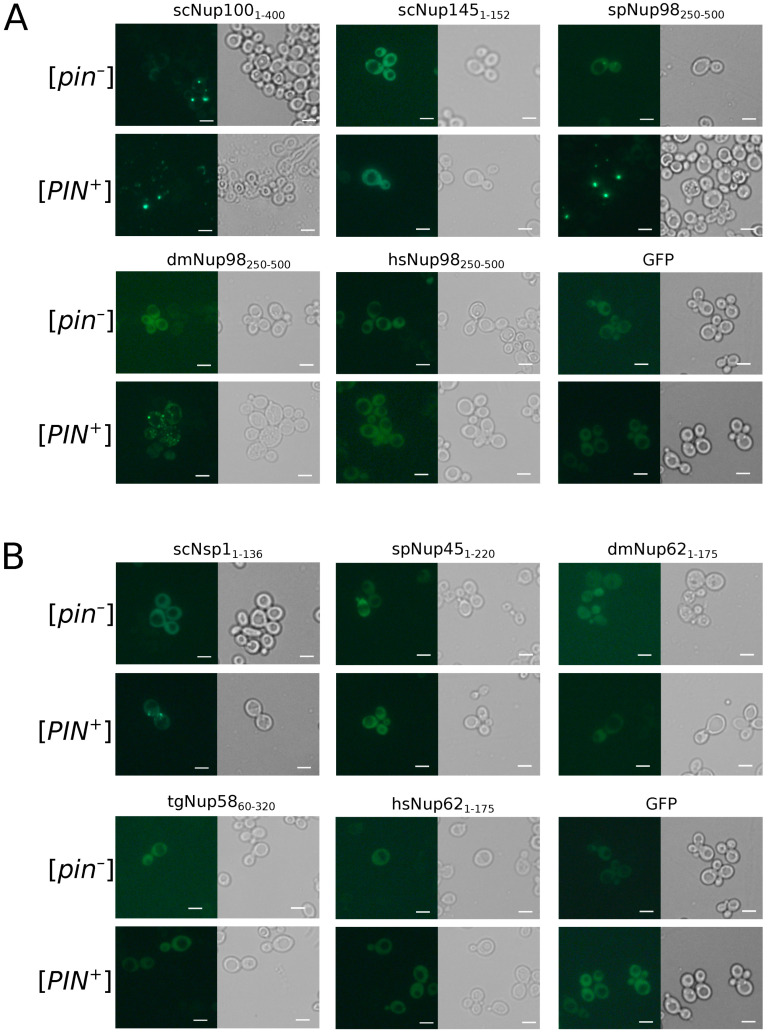
Only few Nups aggregate in yeast cells: The microphotographs of orthologs Nup100 (**A**) and Nsp1 (**B**) protein fused with GFP overproducing nucleoporins’ fragments fused with GFP. GFP alone was taken as negative control. The scale bar equals 25 μm.

To study the aggregation of nucleoporins in the yeast *S. cerevisiae*, we used two isogenic strains, 1-OT56([*PIN^+^*]) and 2-OT56([*pin^−^*]), which differ in the presence of the [*PIN^+^*] prion. Cells of both strains were transformed with plasmids for overproduction of nucleoporins’ fragments fused with GFP. We observed the formation of fluorescent foci for the constructs dmNup98_250–500_, spNup98_250–500_, scNup100_1-400_, and scNSP1_1–175_. Other proteins demonstrated diffuse distribution in the cells, including scNup145_1–152_ and tgNup58_60–320_ (Figure 5), which form amyloid aggregates in bacteria cells. Our results also reveal the necessity of [*PIN^+^*] for Nsp1 aggregation and more efficient aggregation of Nup100. The Rnq1 aggregates had no effects on the aggregation of other analyzed proteins (Table 2).

## 3. Discussion

Nowadays, a large number of amyloids are known among various groups of organisms [6]. Some of these proteins are functional amyloids and are responsible for various biological functions. Remarkably, several of such proteins share their aggregation properties with their orthologs, among them are CPEB [33], FXR1 [9,10], and RHIM-motifs containing proteins [34]. According to the published data, nucleoporins may represent another example of a protein family with conservative amyloid properties.

The human NUP58 protein can form amyloid aggregates in different model systems [19]. Some yeast nucleoporins have been shown to form amyloid aggregates. Namely, the fragment of yeast Nsp1 protein from 1 to 175 amino acids forms detergent resistant aggregates in yeast cells under overproduction, and corresponding aggregates obtained in vitro are stained with ThT [15]. First, 601 amino acids of the protein form hydrogel in vitro [25], which contains interchain β-sheets—the characteristic feature of amyloid aggregates [26]. The same results were obtained for Nsp1_2–277_ [16]. The yeast Nup100_1–592_ fragment was shown to form detergent-resistant aggregates in the cells and ThT-positive aggregates in vitro [15]. Further analysis of this protein demonstrated that shorter fragments (1–200 and 201–400) of the protein are able to aggregate in yeasts, and the Nup100_300–400_ forms fibrils which are stained with ThT [17]. Our data about Nsp1 and Nup100 aggregation are consistent with these facts and provide a new additional evidence for the amyloidogenic properties of yeast Nsp1_1–136_ and Nup100_1–400_: fibrillar morphology of aggregates and Congo Red staining, followed by apple-green birefringence (Figure 3 and Figure 4).

Our bioinformatic analysis revealed that many nucleoporins from different taxonomic groups are potential amyloids (Figure 1). Moreover, for orthologs of Nup49, Nup57, Nup159, and Nsp1, we revealed regions with conservative aggregation-prone properties (Figure 2). However, only two (dmNup98_250–500_ and spNup98_250–500_) out of five nucleoporins whose aggregation had not been studied yet demonstrated amyloid properties in bacteria and yeast cells (Figure 4 and Figure 5). Another two proteins (scNup145_1–152_ and tgNup58_60–320_) aggregated only in C-DAG experiments (Figure 3 and Figure 4). These results do not support the conservatism of amyloid properties of nucleoporins. However, we cannot not exclude that the used yeast and bacterial model systems may not reflect the processes in corresponding organisms. From another point of view, we demonstrated new examples of Nups’ amyloidogenic fragments.

Previously, it was proposed that the amyloid fibrils play a role in the formation of hydrogel by yeast Nup49 and human NUP153 FG-repeat regions [18]. At the same time, it was shown that hydrogels formed by different Nups could not be enriched by β-strand structures, and it was supposed that different types of molecular cohesion may be implicated in selective barrier formation [35]. Moreover, recent studies support that Nups with FG-repeats rather undergo liquid-like phase separation than form stable aggregates [20,21]. Thus, the question about the role of amyloids in nuclear transport is debatable. We suppose that the irreversible aggregation of nucleoporins is rather an abnormal process.

## 4. Materials and Methods

### 4.1. Bioinformatic Analysis

Sets of the Nups (Nsp1, Nup1, Nup2, Nup42, NUP49, NUP50, NUP54, Nup57, NUP58, Nup60, Nup100, Nup153, Nup159) protein orthologs for the Opistokhontha taxonomic group were obtained from the EggNOG orthologs database (access date—28 November 2020) [36]. Sequences were aligned and manually filtered to exclude duplicated, not, or inaccurately annotated sequences or sequences with long indels (more than 20 amino acids) extremely differing from the consensus ones. The amyloid properties were predicted by ArchCandy with a threshold value of 0.575 [22]; unstructured regions were predicted by the IUPred program (with the ‘long’ option and threshold value 0.3) [37,38]. The protein regions with an IUPred score of more than 0.3 were considered as unstructured. A protein or its part was considered amyloidogenic if at least one β-arch (based on ArchCandy predictions) with a score above the threshold was located in an unstructured region. Several taxonomic groups were excluded from the analysis in order to avoid bias in the results of subsequent studies due to overrepresented taxonomic groups. Thus, the filtration was conducted in a way to include only groups, satisfying the following conditions: (i) classes including from 3 to 10 sequences; (ii) if there are more than 10 sequences in the class ten of them ought to be chosen accidentally. Such sampling was repeated 10 times for each orthologs’ dataset. To estimate the conservativity of orthologs’ sequences, alignment with the MUSCLE algorithm was performed (the draft alignment followed by the refinement step). R package muscle was used for automatization [39]. Then, ArchCandy scores were rearranged according to the new alignments’ amino acid positions. If there was a gap in the position of the alignment, the NA value was included into the rearranged ArchCandy resulting table. Except for the conservativity of alignments, the conservatism of amyloidogenic properties (CAP, frequency of cases when corresponding position is located inside the amyloidogenic region) and gaps’ proportions were also estimated. The CAP at a particular alignment position was estimated as the fraction of sequences in which the corresponding amino acid possesses ArchCandy metrics higher than 0.575. The conservatism of amyloid properties in each position of the alignment was evaluated as the fraction of sequences in which the corresponding amino acid is included into the amyloidogenic region. The proportion of gaps was calculated as the fraction of gaps at a particular position of the alignment. This analysis was performed in R [40] with the Biostring package ([41]). The tidyverse package, including the ggplot2, dplyr, and plyr packages, were used for data rearrangement and plotting [42]. All supporting functions are available via github repository.

### 4.2. Plasmid Construction

For yeast nucleoporins’ gene fragment cloning, yeast (*S. cerevisiae*) genomic tiling collection [43] or total *S. cerevisiae* genome DNA (strain OT56) were used. The total RNA was extracted from 972 *S.pombe* strain, human cell line IMR-32 (a gift from D.V. Kachkin), genetic line *D. melanogaster* (DGRP-859), and a sample of *T. guttata* (a gift from S.A. Galkina). First-strand cDNA was synthesized by the RevertAid RT Kit (Thermo Scientific, K1691), as described in the protocol.

The coding sequences of Nups fragments were PCR-amplified with primers containing *att*B sites (Appendix A Table A1). The PCR products of corresponding sequences flanked with *att*B sites were inserted into pDONR221-ccdB (Thermo Scientific, Waltham, MA, USA), 12536017) by BP reaction (BP Clonase™ II Enzyme mix, Thermo Scientific, Waltham, MA, USA). Obtained plasmids were verified by sequencing in the resource center “Molecular and Cell Technologies” of Saint Petersburg State University. The coding sequences were cloned to the pVSGW-ccdB [19] and pAG416GPD-EGFP-ccdB (a gift from Susan Lindquist (Addgene plasmid 14316 http://n2t.net/addgene:14316 accessed on 13 July 2022)). Obtained plasmids were used for overproduction of Nups fragments in the C-DAG and yeast model systems, respectively.

### 4.3. Microbiological Procedures and Strains

Standard microbiological approaches and media were used for all manipulations with bacteria and yeast [44,45]. Yeast transformation was performed as described previously [45]. *Escherichia coli* strains TOP10 (Invitrogen) and DH5α [44] were used for cloning; DB3.1 (Thermofisher) was used for production of plasmids with *ccdB* cassette. The strain 1-OT56 with genotype *MAT*a, *ade1-14(UGA) trp1-289(UAG) ura3-52 his3-200 leu2-3, 122* [*psi^–^*] [*PIN^+^*] and strain 2-OT56 with genotype *MAT*a, *ade1-14(UGA) trp1-289(UAG) ura3-52 his3-200 leu2-3, 122* [*psi^–^*] [*pin^–^*] were used for experiments assessing protein aggregation [46].

### 4.4. Fluorescent Microscopy

Transformants, selected on synthetic complete medium lacking uracil (SC-Ura), were grown in the liquid selective media at 30 °C to logarithmic phase. Cells were gently pelleted (2000–3000 rpm), washed from the medium, and resuspended in water. Fluorescence was analyzed using a ZeissAxioScope.A1 wide-field fluorescence microscope. Images were taken with a ZEISS Axiocam 506 color camera using ZEISS ZEN lite 3.0 software.

### 4.5. C-DAG Experiments

The experiments in the C-DAG system cells of *E. coli* strain VS39 [27] were transformed with target plasmids, and transformants were selected on medium supplemented with ampicillin (100 μg/mL) and chloramphenicol (25 μg/mL) at 37 °C [44]. The overnight bacterial culture was diluted 100-fold and grown for one hour. The obtained cultures were plated on a series of media: CR-inducing plate L-arabinose (0.1% (*w*/*v*), IPTG (2 mM), Congo Red dye (10 μg/mL), ampicillin (200 μg/mL), and chloramphenicol (25 μg/mL)); plate with inductors (L-arabinose 0.1% (*w*/*v*), IPTG (2 mM), ampicillin (200 μg/mL), and chloramphenicol (25 μg/mL)); and a control plate containing only antibiotics. Plates were incubated at 37 °C for 3 days [27]. Plasmids pVS-GW-Sup35NM and pVS-GW-Sup35M were used as positive and negative controls, respectively [19].

Samples for transmission electron microscopy were prepared by applying 5 µL of bacteria cell suspension from a CR-inducing plate on a formvar-coated grid, followed by washing with distilled water and drying. The samples were stained with the dye for 30 s. The excess of the uranyl acetate was removed with incubation in distilled water for 30 s. Jeol JEM-2100 transmission electron microscope was used for the subsequent analysis. Samples for polarisation microscopy were prepared as follows. A total of 20 μL of the cell’s suspension was applied on a slide and dried. Then, the cells were analyzed with an inverted Leica DMI6000 microscope.

### 4.6. Statistical Analysis

Fisher’s exact test [47] was used to compare the proportion of cells with a particular phenotype. All calculations were performed using the R software [40].

## Figures and Tables

**Table 1:** Proteins, corresponding species, and fragments used in the study.

Protein	Species	Fragment	Name
Nsp1	*S. cerevisiae*	1–136	scNsp1_1–136_
Nup100	*S. cerevisiae*	1–400	scNup100_1–400_
Nup145	*S. cerevisiae*	1–152	scNup145_1–152_
Nup45	*Schizosaccharomyces pombe*	1–220	spNup45_1-220_
Nup58	*Taeniopygia guttata*	60–320	tgNup58_60–320_
Nup62	*D. melanogaster*	1–175	dmNup62_1–175_
NUP62	*Homo sapiens*	1–175	hsNup62_1–175_
Nup98	*S. pombe*	250–500	spNup98_250–500_
Nup98	*D. melanogaster*	250–500	dmNup98_250–500_
NUP98	*H. sapiens*	1–250	hsNup98_1–250_

**Table 2:** The [*PIN^+^*] factor increases the frequency of yeast nucleoporins aggregation. The table contains information about the proportions of cells with fluorescent aggregates. Fisher’s exact test was used to compare the frequencies of cells with aggregates. Results of three independent replicates are presented (see Appendix A Table A2 for raw data).

Protein	% of Cells with Fluorescent Foci in [*PIN^+^*] Strain	% of Cells with Fluorescent Foci in [pin^−^] Strain	Statistical Significance (*p*-Value < 0.05)
dmNup98_250–500_	2.75 ± 0.622	1.83 ± 0.525	no
spNup98_250–500_	3.50 ± 0.675	3.41 ± 0.699	no
hsNup98_1–250_	1.29 ± 0.455	0.48 ± 0.28	no
hsNup62_1–175_	0.0 ± 0.498	0.7 ± 0.312	no
dmNup62_1–175_	0.29 ± 0.211	0.0 ± 0.161	no
spNup45_1–220_	0.0 ± 0.16	0.16 ± 0.165	no
tgNup58_60–320_	0.0 ± 0.152	0.0 ± 0.139	no
scNsp1_1–136_	4.99 ± 0.835	0.0 ± 0.162	yes
scNup100_1–400_	52.11 ± 2.055	31.94 ± 1.687	yes
scNup145_1–152_	0.32 ± 0.227	0.16 ± 0.162	no

## Data Availability

Data avilable on request, and All supporting functions are available via github repository.

## Abbreviations

The following abbreviations are used in this manuscript:

C-DAG	curli-dependent amyloid generator
FG-repeats	phenylalanine–glycine-repeat
NPC	nuclear pore complex
Nups	nucleoporins

## Appendix A

**Table A1:** Primers used in work: Bold letters indicate *att*B recombination sites.

Primers Name	Sequence	T Melt (°C )
*att*b1-Nup100-1-400-F	**GGGGACAAGTTTGTACAAAAAAGCAGGCT**CAACAATGTTTGGCAACAATAGACCAATGTTTGG	61
*att*b2-Nup100-1-400-R(stop)	**GGGGACCACTTTGTACAAGAAAGCTGGGT**CGTTATGCTGGTTTGGCTCCAAACAAACCTGTAG	61
*att*b1-Nup145-1-152-F	**GGGGACAAGTTTGTACAAAAAAGCAGGCT**CAACAATGTTTAATAAAAGTGTAAATAGTGG	59
*att*b2-Nup145-1-152-R(stop)	**GGGGACCACTTTGTACAAGAAAGCTGGGT**CGTTAATTTTGCGTGGTAGAAGTTATATTG	59
*att*b1-Nsp1-1-136-F	**GGGGACAAGTTTGTACAAAAAAGCAGGCT**CAACAATGAACTTCAATACACCTCAACAAAACAAAACGCCC	63
*att*b2-Nsp1-1-136-R(stop)	**GGGGACCACTTTGTACAAGAAAGCTGGGT**CGTTAATTGCTATTAGTACTGTTATTGAATAAGGATG	63
*att*B1-hsNup98-250-F	**GGGGACAAGTTTGTACAAAAAAGCAGGCT**CAGCATATGGTCAGAACAAAACTGCC	61
*att*B2-hsNup98-stop-499-R	**GGGGACCACTTTGTACAAGAAAGCTGGGT**CGTTAAACAGCCTGCTGGGCAGCAG	61
*att*B1-spNup98-250-F	**GGGGACAAGTTTGTACAAAAAAGCAGGCT**CAAATCAAGGACGACGTTTTGGC	60
*att*B2-spNup98-stop-499-R	**GGGGACCACTTTGTACAAGAAAGCTGGGT**CGTTAATTAGCATTTTGCCCAAAAGAAAAACCTCC	60
*att*B1-tgNup58-60-320-F	**GGGGACAAGTTTGTACAAAAAAGCAGGCT**CAATGGGACTTAATTTTGGAGCTCTGGGCTCTTCC	60
*att*B2-tgNup58-60-320-R	**GGGGACCACTTTGTACAAGAAAGCTGGGT**CGTTATAGCTCCTGTGCAGTTTCCACTTTCAGCTTGTC	60
*att*B1-spNup45-1-F	**GGGGACAAGTTTGTACAAAAAAGCAGGCT**CAATGTTCGGGTTAAATAAAACACCC	60
*att*B2-spNup45-220-R	**GGGGACCACTTTGTACAAGAAAGCTGGGT**CGTTATGTTGACGGTTGCTGCTG	60
*att*B1-dmNup62-1-F	**GGGGACAAGTTTGTACAAAAAAGCAGGCT**CAATGGTATTCCAGTTGCCAACAAC	61
*att*B2-dmNup62-175-R	**GGGGACCACTTTGTACAAGAAAGCTGGGT**CGTTACTGCGTGGAGGCTATGGC	61
*att*B1-dmNup98-250-F	**GGGGACAAGTTTGTACAAAAAAGCAGGCT**CATGCGGGCGCAAGGGTCCAC	60
*att*B2-dmNup98-500-R	**GGGGACCACTTTGTACAAGAAAGCTGGGT**CGTTAGCTGGTGGCTCCAAAACC	60
*att*B1-hsNUP62-1-F	**GGGGACAAGTTTGTACAAAAAAGCAGGCT**CAATGAGCGGGTTTAATTTTGGAGG	59
*att*B2-hsNUP62-175-R	**GGGGACCACTTTGTACAAGAAAGCTGGGT**CGTTATGAGCCAATGTTGAAACCGG	59

**Table A2:** Amount of cells with aggregates in strains with [*PIN^+^*] and [*pin^−^*]. ***—*p*-value < 0.001 (Fisher’s exact test).

Protein	Amount of Cells with Diffuse Glow	Amount of Cells with Fluorescence Foci	% of Cells with Fluorescence Foci	[*PIN*^+^] Status	*p*-Value
dmNup98_250–500_	672	19	2.75 ± 0.622	[*PIN^+^*]	0.2812
	642	12	1.83 ± 0.525	[*pin^−^*]	
spNup98_250–500_	716	26	3.50 ± 0.675	[*PIN^+^*]	1
	651	23	3.41 ± 0.699	[*pin^−^*]	
hsNup62_1–175_	620	0	0.0 ± 0.498	[*PIN^+^*]	0.06514
	709	5	0.7 ± 0.312	[*pin^−^*]	
dmNup62_1–175_	666	2	0.29 ± 0.211	[*PIN^+^*]	0.5003
	619	0	0.0 ± 0.161	[*pin^−^*]	
spNup45_1–220_	623	0	0.0 ± 0.16	[*PIN^+^*]	0.4923
	603	1	0.16 ± 0.165	[*pin^−^*]	
tgNup58_60–320_	653	0	0.0 ± 0.152	[*PIN^+^*]	1
	718	0	0.0 ± 0.139	[*pin^−^*]	
hsNup98_1–250_	610	8	1.29 ± 0.455	[*PIN^+^*]	0.2245
	615	3	0.48 ± 0.28	[*pin^−^*]	
scNsp1_1–136_	647	34	4.99 ± 0.835	[*PIN^+^*]	2.17 × 10^−10^ ***
	615	0	0.0 ± 0.162	[*pin^−^*]	
scNup100_1–400_	283	308	52.11 ± 2.055	[*PIN^+^*]	6.864 × 10^−14^ ***
	520	244	31.94 ± 1.687	[*pin^−^*]	
scNup145_1–152_	620	2	0.32 ± 0.227	[*PIN^+^*]	1
	615	1	0.16 ± 0.162	[*pin^−^*]	
